# Reliability Study of Inertial Sensors LIS2DH12 Compared to ActiGraph GT9X: Based on Free Code

**DOI:** 10.3390/jpm12050749

**Published:** 2022-05-05

**Authors:** Jaime Martín-Martín, Ariadna Jiménez-Partinen, Irene De-Torres, Adrian Escriche-Escuder, Manuel González-Sánchez, Antonio Muro-Culebras, Cristina Roldán-Jiménez, María Ruiz-Muñoz, Fermín Mayoral-Cleries, Attila Biró, Wen Tang, Borjanka Nikolova, Alfredo Salvatore, Antonio I. Cuesta-Vargas

**Affiliations:** 1 Biomedical Research Institute of Málaga (IBIMA), 29010 Málaga, Spain; jaimemartinmartin@uma.es (J.M.-M.); detorres.irene@gmail.com (I.D.-T.); adrianescriche@gmail.com (A.E.-E.); mgsa23@uma.es (M.G.-S.); amuro@uma.es (A.M.-C.); cristina.roldan005@gmail.com (C.R.-J.); marumu@uma.es (M.R.-M.); fermin.mayoral.sspa@juntadeandalucia.es (F.M.-C.); 2 Legal and Forensic Medicine Area, Department of Human Anatomy, Legal Medicine and History of Science, Faculty of Medicine, University of Málaga, 29071 Málaga, Spain; 3 Department of Computer Science and Programming Languages, Higher Technical School of Computer Engineering, University of Málaga, 29071 Málaga, Spain; ariadnapartinen@lcc.uma.es; 4 Physical Medicine and Rehabilitation Unit, Regional Universitary Hospital of Málaga, 29010 Málaga, Spain; 5 Department of Physiotherapy, University of Málaga, 29071 Málaga, Spain; 6 Department of Nursing and Podiatry, University of Málaga, 29071 Málaga, Spain; 7 Mental Health Unit, Regional Universitary Hospital of Málaga, 29010 Málaga, Spain; 8 ITWare, 1117 Budapest, Hungary; attila.biro@itware.hu; 9 Faculty of Science and Technology, Bournemouth University, Bournemouth BH12 5BB, UK; wtang@bournemouth.ac.uk; 10 Arthaus, Production Trade and Service Company Arthaus Doo Import-Export Skopje, 1000 Skopje, North Macedonia; borjanka@arthaus.mk; 11 Sensor ID Snc., 86021 Boiano, Italy; alfredo.salvatore@sensorid.it; 12 School of Clinical Science, Faculty of Health Science, Queensland University Technology, Brisbane 4000, Australia

**Keywords:** inertial sensors, R code, open-source code, reliability, functional assessment

## Abstract

The study’s purpose was to assess the reliability of the LIS2DH12 in two different positions, using the commercial sensor Actigraph GT9X as a reference instrument. Five participants completed two gait tests on a treadmill. Firstly, both sensors were worn on the wrist and around the thigh. Each test consisted of a 1 min walk for participants to become accustomed to the treadmill, followed by a 2 min trial at ten pre-set speeds. Data from both sensors were collected in real-time. Intraclass correlation coefficient (ICC) was used to evaluate the equality of characteristics obtained by both sensors: maximum peaks, minimum peaks, and the mean of the complete signal (sequence of acceleration values along the time) by each axis and speed were extracted to evaluate the equality of characteristics obtained with LIS2DH12 compared to Actigraph. Intraclass correlation coefficient (ICC) was extracted, and a standard deviation of the mean was obtained from the data. Our results show that LIS2DH12 measurements present more reliability than Actigraph GT9X, ICC > 0.8 at three axes. This study concludes that LIS2DH12 is as reliable and accurate as Actigraph GT9X Link and, therefore, would be a suitable tool for future kinematic studies.

## 1. Introduction

Inertial Measurement Units (IMUs) are equipped with an accelerometer that measures linear accelerations and a gyroscope that measures the angular velocity of the device recorded in the three directions of space [1]. Furthermore, some of them also include a magnetometer (measure the direction or strength); an angular rate and gravity (MARG) sensor module for measuring 3 degrees of freedom orientations in real-time [2]. These systems are validated against 3D movement capture devices [3]. Its use is extended to the analysis of human movement [4]. These devices have been used in the health field to validate symptoms of patients who have Parkinson’s disease [5], and the physical activity of patients in a wheelchair have been quantified [6] to calculate energetic waste [7]. Furthermore, sensors have been used as a rehabilitation instrument accompanied by other peripheral devices [8].

There is an extensive range of commercially available devices, but not all are validated and suitable for recording human motion [9]. The reliability of the obtained data is influenced by the sensor’s accuracy, the components that enclose the sensor, the position of the sensor, and the data processing [10,11]. The reliability evaluation should be applied to protocols, which can be reproduced later [12,13]. In this regard, Actigraph GT3X (Pensacola, FL, USA) [14] is one of the most used widely used thanks to its demonstrated reliability and straightforward operation [15].

Actigraph (ActiGraph LLC., Pensacola, FL, USA) is one of the leading commercially available devices [15]. It has been used as a reference to validate other devices, such as activity monitors, to determine physical activity [16]. Likewise, activPAL was compared to measure children’s sedentary behaviour aged 2–3 years [17]. Additionally, Actigraph was used relative to FitbitZip to measure steps made by patients suffering rheumatic polymyalgia [18]. In every case, results were derived from the pre-processed device’s data and not from the raw data extracted by the sensor.

There are currently reliable, low-cost devices (below $50) and validated alternatives to record steps made and physical activity [19]. The LIS2DH12 (STMicroelectronics; Shanghai, China) chip is an ultralow consumption, a high-performance accelerometer that records in three axes of space [20] and is compatible with Arduino systems [21]. LIS2DH12 is a novel sensor that has not been used in previous studies. Validating the device will make it useful for specific future applications like kinematic analysis or activity tracker. The advantage of the device, once validated, is a low cost and low consumption sensor that would allow the free design of third-party applications, unlike commercial sensors. Its acceleration measurements have not been compared with other reference devices, such as Actigraph, to evaluate the reliability of its measurements.

Therefore, this study aimed to assess the reliability of the chipset LIS2DH12 relative to the internal accelerometer of Actigraph GT9X Link as an instrument to measure accelerations produced during human movements.

## 2. Materials and Methods

The present study is based on a reliability study of inertial sensor Lis2DH12 chipset (“STMicroelectronics”) (equipped on a SensorID device), compared with the measurements given by the commercial device Actigraph GT9X Link (“ActiGraph Link|ActiGraph”). Actigraph GT9X Link is reliable to assess sedentary behavior and physical activity based on raw data or energy expenditure [22,23]. To obtained data, the selected movement was the gait. A protocol of increasing walking/running speeds, recorded on a treadmill, was used.

### 2.1. Equipment

The LIS2DH12 inertial sensor has a sampling frequency of 1 Hz to 5.3 kHz. The sensor measures the acceleration in three axes (X, Y, Z) with a values range of ±16 g. The sensor is assembled on a mainboard designed by SensorID (“Sensor ID-Proximity Wireless Technologies Made in Italy”). The device’s size is a circumference with a radius of 4.5 cm and 1 cm thick. The software was designed by SensorID, with a preset sampling frequency of 30 Hz to record the data. The data were extracted directly in raw format. This software captures sensor data in m/s^2^ and mg (gravitational unit value) units. The sensor signs the gravitational acceleration of Earth (9.8 m/s^2^). The data captured by the sensor are sent to the computer in real-time by a 5.0 Bluetooth protocol.

Actigraph GT9X Link has a sampling frequency range of 30 Hz to 100 Hz with a range of ±16 g. The dimensions of the device are 3.5 × 3.5 × 1 cm. Actigraph is available to provide actionable data on a scale of 0 to 15,000 count/min. The sensor data are recorded in the internal memory and later transferred to the computer via a micro-USB. The data used, which Actigraph GT9X Link gives, are the raw data from accelerations measured during the movement. The movement chosen was the human gait.

### 2.2. Data Collection

The clocks of both sensors were synchronized with the computer’s clock to ensure simultaneous recording. Both sensors were calibrated before starting to measure, placing both sensors on the same smooth surface. Two experimental setups/device locations were explored during the study. In one of them, both sensors (Actigraph GT9X Link and LIS2DH12) were placed simultaneously (both sensors overlapping) on the participant’s right wrist. Actigraph GT9X Link was used with its wristwatch accessory, with LIS2DH12 positioned with a stretchable belt. 

In the second set, both sensors were placed on the participant’s thigh using a stretchable belt. This second set was carried out after the first one had finished evaluating validation at both positions. Both positions were chosen because the main objective was to collect data which could be compared. Therefore, the wrist and thigh are a good option, given that they have easy access and handling.

Five healthy adults participated in the reliability study. Two tests were carried out, each of 20 min in total; this sample size gives a large amount of data because it is a record of 200 min with a sampling frequency of 30 Hz, recording approximately 360,000 values. Actigraph GT9X Link and LIS2DH12 were placed on the right wrist and thigh, not simultaneously. The participant wore sportswear, adequate footwear, and short sleeves. Data were collected while walking and running at different speeds on a treadmill, with each wrist and thigh position evaluated separately. For each of the speeds, ten measurements were obtained with the two sensors together (five on the wrist and five on the thigh). This assumes a Confidence Level of 80% and a margin of error of 21%.

The predetermined speeds were: 1.4 km/h, 2.9 km/h, 4.3 km/h, 5 km/h, 6 km/h, 6.5 km/h, 7.0 km/h, 8.0 km/h, 9.0 km/h and 10.0 km/h. These speeds were representative a range of different subject types: subjects who were dependent, hospitalized, needing rehabilitation, discharged, regular patients and healthy subjects [24]. Participants started walking on the treadmill at a pre-set speed for one minute to adapt to rhythm, subsequently starting the recording of measured data for two minutes. Each speed for walking and running was measured separately for two minutes. Participants were asked to carry out natural movements.

### 2.3. Data Processing

**Figure 1:**
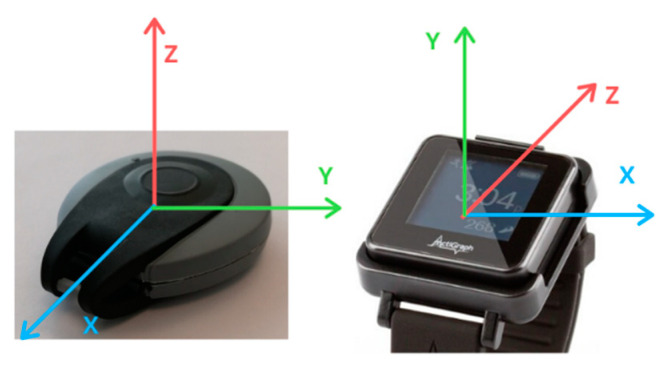
Reference system by the manufacturer.

The spatial reference system of sensor LIS2DH12 was adapted to the spatial reference system of Actigraph GT9X Link. If the spatial reference system of Actigraph GT9X Link is set as our reference system, the axes of LIS2DH12 coincide with Actigraph GT9X Link as showed in Figure 1: x-axis of LIS2DH12 is equal to z-axis of Actigraph GT9X Link, y-axis of LIS2DH12 coincides with the x-axis of Actigraph GT9X Link, and z-axis of LIS2DH12 is equivalent to y-axis of Actigraph GT9X Link (Figure 1). The orientation in repose was analyzed, and then both sensors were always put in the same position. The change of basis matrix (*RL,A*), which can be used to transform data from one reference frame to the other, is provided below:

$$R_{L,A}=\;\left[\begin{array}{ccc} 0 & 1 & 0\\ 0 & 0 & 1\\ -1 & 0 & 0 \end{array}\right]\;R_{L,A}^{-1}=R_{A,L}=\;\left[\begin{array}{ccc} 0 & 0 & -1\\ 1 & 0 & 0\\ 0 & 1 & 0 \end{array}\right]$$

Actigraph GT9X Link reference system $\begin{array}{c} R_{A,L}\\ ⇄\\ R_{L,A} \end{array}$ LIS2DH12 reference system.

Data processing was carried out with RStudio software version 1.2.5033, under R 3.6.2 (R Project, 2002). The codes generated for analysis can be seen in Appendix A.

One hundred files (five participants, ten speeds, and two positions to validate) in “.csv” format, belonging to Actigraph GT9X Link sensor, and another one hundred from LIS2DH12 were processed for analysis. One file was defective because it was recorded erroneously, meaning the file from the second subject at 1.4 km/h from Actigraph GT9X Link was rejected, along with its counterpart from LIS2DH12. Therefore, 198 files were analyzed in total.

Files were read with R and adapted so the formats of both sensors were the same. LIS2DH12 records the acceleration in thousandths of g-force (mg), and Actigraph GT9X Link uses g-force (g) units. Acceleration units were converted into multiples of g to enable comparison.

**Figure 2:**
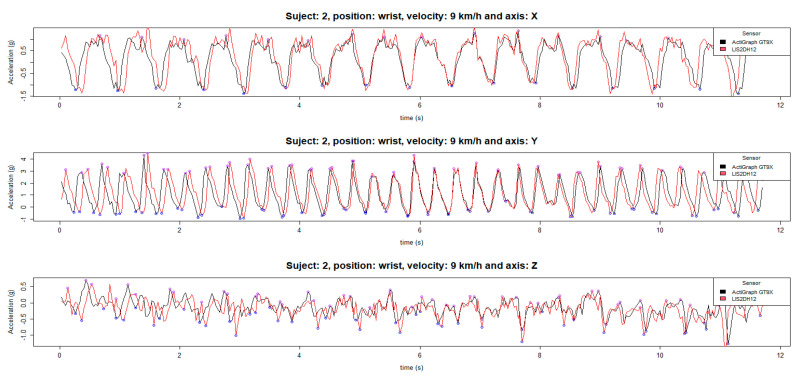
Data sensors ActiGraph GT9X Link and LIS2H12 x-axis, y-axis and z-axis at 9 km/h on the wrist.

Two functions were designed to import data from different signed subjects to R (readSensorIdData() y readActigraphData(), Appendix A). Afterwards, 198 files were read to collect in “RData” format for subsequent use (Figure 2).

Comparison tables were drafted based on the data collected in R. 

Both sensors were configured to have a sampling frequency of 30 Hz, with two minutes of gait test equivalent to 3600 samples, but there was a loss of around 50 random samples in data from LIS2DH12, due to the transmission of data by Bluetooth. Two recorded signals of both sensors are shown in Figure 2, where the gap between signals is not homogeneous throughout the entire signal, but first, they are shifted, then they are overlapped and shifted again. This fact occurs at all the times recorded. Therefore, comparison sample-by-sample was not possible, meaning that we had to compare both recorded signals using the mean of the relevant features.

After the process, the features/measurements of interest extracted from the data were the mean of maximum peaks, mean of minimum peaks (valleys), and signal mean extracted from each axis and sensor.

A predefined function of R: findpeaks() [25] was used to extract the maximum and minimum peaks of the signal, straightaway we calculated, using the mean() function, the mean of these peaks and the mean of the signal. In total, eighteen columns were obtained to compare each one (nine from the LIS2DH12 sensor and 9 from Actigraph GT9X Link).

### 2.4. Statistical Analysis

A statistical analysis was carried out in RStudio software with version 1.2.5033, under R 3.6.2. The intraclass correlation coefficient (ICC) was calculated to determine the reliability of LIS2DH12 compared to Actigraph GT9X Link. A descriptive analysis (mean and standard deviation) was developed.

Variability is reflected by standard deviation, allowing quantification of their differences; if dataset´s the standard deviation is a low value (relative to mean), the data are more similar. If it is equal to zero, the samples are identical.

ICC analysis allowed quantification of the variance between measurements of phenomena measured with different raters (in this case, two instruments, Actigraph GT9X Link and LIS2DH12). ICC values can fluctuate between 0 and 1, with 0 indicating that these measurements are not in agreement and 1 that these measurements are reliable. These values may be interpreted as excellent reliability if ICC ≥ 0.75, ICC between 0.4 and 0.74 indicates fair-to-high reliability, and ICC ≤ 0.39 is interpreted as poor reliability [26]. ICC was calculated with a level of significance of 0.05 and a confidence interval of 95%, a “two-way” model, because in this assay, each subject was measured by the same set of known raters, that is, Actigraph GT9X Link and LIS2DH12, considering subjects and rater’s random effects [27]. Absolute agreement analysis was used; as the study concerns if different raters assign the same score to the same subject. This agreement had two options type: “single measurements” and “average measurements”, depending on calculating the reliability by taking single measurements or whether calculating the average of the measurements from multiple raters [26,28]. 

The statistical analysis was applied to the features/measurements of interest: the mean of the maximum peaks, the mean of minimum peaks and the signal´s mean, recorded in each of the three axes (18 in total).

ICC was calculated by each measurement from both sensors, e.g., the value obtained from Actigraph GT9X Link as the mean of maximum peaks (measurement) of every subject at 1.4 km/h in x-axis was compared to its counterpart LIS2DH12 sensor to obtain ICC values.

### 2.5. Ethical Aspects

This study belongs to the European Union’s Horizon 2020 research and innovation program under the Marie Sklodowska-Curie grant agreement No. 823871 (iGame), and it was approved by the Ethics Committee of the Malaga Provincial Research of the Andalusian Health Service. The study code was: RCT-iGAME. All participants accepted and signed the informed consent following the principles established in the Declaration of Helsinki.

## 3. Results

Five healthy adults participated in the reliability study, whose average and standard deviation of BMI (Body Mass Index) was 21.43 kg/m^2^ ± 2.23 kg/m^2^, indicating healthy weights, and their ages oscillated between 22 and 33 years old. 

Descriptive and statistical analysis was completed using ICC, “single measurements” and “average measurements” options, as reported before, to compare the features/measurements of interest; that is, mean of maximum peaks, mean of minimum peaks and the mean of the complete signal from each axis and sensor.

Table 1, for wrist position, and Table 2, for thigh position, are reported the ICC values obtained from comparing the data of all subjects for each measurement of interest along with their standard deviations and grouped by speed (km/h) and axis. 

Concerning Table 1, wrist position, the average of ICC values, with “single measurements” and “average measurements” options, obtained for each feature/measurement (mean of maximum peaks, mean of minimum peaks and mean of complete signal) oscillated between 0.76 and 0.90. The mean of ICC, “single measurements” and “average measurements, is 0.793/0.863, this range indicates excellent reliability. The three best values of ICC, “single measurements” and “average measurements”, were 0.999/0.999, corresponding to mean of maximum peaks of z-axis at 9 km/h on wrist position (Table 1); and 0.996/0.999, corresponding to the mean of z-axis at 9 km/h in wrist position (Table 1). In contrast, the three worst ICC values were 0.104/0.188, corresponding to the mean of x-axis at 10 km/h on wrist position, and 0.273/0.429, corresponding to the mean of minimum peaks of the y-axis at 1.4 km/h on wrist position (Table 1).

For the thigh position, Table 2, the ICC values oscillated between 0.78 and 0.92, and the mean of ICC, “single measurements” and “average measurements, was 0.834/0.895, indicative of excellent reliability. The best ICC values were 0.998/0.999, corresponding to the mean of minimum peaks of the z-axis at 8 km/h in the thigh position (Table 2). The worst values for ICC were 0.144/0.251, corresponding to mean of minimum peaks of the y-axis at 1.4 km/h in the thigh position (Table 2). Therefore, the order of ICC obtained was 0.8, meaning excellent reliability [25].

Table 3 is a summary that synthesizes the information from Table 1 and Table 2. In this table, concerning the wrist, it could be observed that the z-axis had the highest ICC values, 0.93 (“single measurements” option) and 0.96 (“average measurements” option), and also the lowest standard deviation, 0.33. Speed 9 km/h had the best ICC values of all speeds, 0.91 and 0.95. In contrast, at low speeds (1.4 and 2.9 km/h), there are poor ICC values, around 0.6, and at speeds between 4.3 km/h and 8 km/h, ICC values obtained are higher than 0.75, being good reliability.

However, the ICC values were generally higher in the thigh position than in the wrist position. The axis with the best ICC values was axis Z, 0.87, and 0.93, “single measurements” option and “average measurements” option. Meanwhile, the speed with the greatest ICC values was 10 km/h, with ICC values of 0.90 and 0.94. The ICC values obtained were above 0.8 for all rows, but two speeds 1.4 and 2.9 km/h showed lower values of ICC in wrist. Therefore, the thigh position showed excellent reliability (Table 3). 

## 4. Discussion

The different functions designed in R (Annex 1) allowed the unification of raw data from both sensors, Actigraph GT9X Link and LIS2DH12. This unification improved data flow. The subsequent processing of data (structure, magnitude, type of data, format, orientation of axes, etc.) allowed to establish a semiautomatic system to process all dataset together.

The most relevant features/measurements of the signal were maximum peaks, minimum peaks (valleys) and the mean of the complete signal. R functions extracted these features (findpeaks() and mean() [25]). Based on these features, a new database was generated with the values of each participant’s, mentioned as features, grouped by speed (Annex 1, getInfobySubject() function). An ICC value between a measurement obtained from LIS2DH12 and one obtained from Actigraph GT9X Link, was calculated to quantify the convergence of data (Annex 1 getICCmatrix() function). 

The results obtained in this study, attending to the ICC values (“single measurements” and “average measurements”), suggest that the reliability of LIS2DH12 is appropriate. Therefore, the LIS2DH12 sensor could be an alternative for use in kinematics studies, considering its measurements are very similar to the measurements obtained from the commercial sensor Actigraph GT9X Link and its cost is much lower.

Previous studies had analyzed the reliability of other inertial sensors [29]. This study compared an in-house sensor, VetSens, to Actigraph GT3X to evaluate animals’ physical activity by recording acceleration from both sensors. These results show an ICC value above 0.90, meaning excellent reliability. These results are aligned with that obtained in the present study: ICC values oscillated between 0.76–0.90 for wrist position (Table 1) and 0.78–0.92 for thigh position (Table 2). The mean of ICC “single measurements”/“average measurements”, 0.793/ 0.863 for wrist position (Table 1), and 0.834/0.895 for thigh position (Table 2). The thigh position had slightly better results. It could be because the fastening in the thigh was greater than in the wrist, where the LIS2DH12 sensor could have a little more movement. Additionally, higher speed had better results than lower speed. There are two possible explanations: the first could be that a rude movement is slightly noisier, which masks the differences between both signals. The second could be that run has more harmonic movement, so the signals are better recorded without noise.

Likewise, the results obtained by inertial sensors had also been compared with camera motion analysis [28]. An ICC value of approximately 0.90 for gait was observed. From the comparison of both sensors, the overall ICC obtained in this study was 0.85. Therefore, both results, 0.90 and 0.85, are not very distant, demonstrating that the chosen method is an excellent option to validate measurements in this type of motion. Moreover, thanks to this study [29], it is demonstrated that LIS2DH12 gives significantly valuable results.

The sample size can seem small, with five subjects. However, two tests were carried out, 40 min in total for each participant, and 200 min were recorded. Thus, upon completion of all tests, approximately 360.000 values were recorded. This sample size (5 subjects make the test twice) defines a confidence level of 80% and a margin error of 20%.

One of the main limitations is that although data from LIS2DH12 are recorded in “.csv” format, the generated tables are not well defined. Neither the reader of R nor Excel can separate the columns correctly. This means the files must be manually adjusted before processing the data, adapting the dataset, and then, with R, the files can be read by the read_xlsx() predefined function.

Another relevant aspect is the loss of information in data transmission by Bluetooth. Both sensors were configured to have a sampling frequency of 30 Hz, with two minutes of gait test being equivalent to 3600 samples. There was a loss of around 50 random samples in data from LIS2DH12. Therefore, comparison is not possible instant by instant, meaning comparison must be made using the mean of the relevant features. Nonetheless, as shown in Figure 2, LIS2DH12 can faithfully reproduce the data collected by Actigraph GT9X Link.

Accordingly, the present study can conclude that R and the generation of codes and functions allow a more desirable data flow, improving their processing and comparison.

## 5. Conclusions

According to the result reported in the previous sections, we can conclude that the LIS2DH12 sensor could be an alternative device in kinematic studies, considering that its reliability is excellent, attending to the mean of ICC (model “two-way” and definition of absolute agreement) obtained, which is above 0.8.

In addition, the LIS2DH12 has desired technical characteristics for a sensor: it allows the data to be collected and processed with open-source software, enriching the scientific community; it has a small size and low weight, providing a portable option; it gives reliable and verified acceleration results (compared to validated systems such as Actigraph GT9X Link), and its cost is more affordable than other commercial sensors, so LIS2DH12 could be a good option to use in acceleration studies where the conditions of the assays would be suitable to these characteristics.

## Figures and Tables

**Table 1:** ICC values and standard deviation grouped by speed (km/h) and axis, in wrist position.

ICC WRIST 95%		Mean Peaks Values				Mean Minimum Values				Mean Values				By Rows			
Km/h	Axis	Single ICC	Average ICC	Average Peaks (g)	SD (g)	Single ICC	Average ICC	Average Valleys (g)	SD (g)	Single ICC	Average ICC	Average (g)	SD (g)	Mean Single ICC	Mean Average ICC	Mean Average (g)	Mean SD (g)
1.4	X	0.648	0.786	−0.848	0.021	0.387	0.558	−1.088	0.020	0.584	0.737	−0.941	0.020	0.540	0.694	−0.959	0.020
	Y	0.533	0.696	0.355	0.069	0.273	0.429	0.175	0.065	0.373	0.543	0.264	0.066	0.393	0.556	0.265	0.067
	Z	0.910	0.953	0.177	0.034	0.876	0.934	−0.009	0.041	0.893	0.943	0.085	0.037	0.893	0.943	0.084	0.037
2.9	X	0.581	0.735	−0.749	0.044	0.449	0.620	−1.250	0.061	0.424	0.596	−0.935	0.043	0.485	0.650	−0.978	0.049
	Y	0.029	0.056	0.428	0.173	0.507	0.673	0.107	0.117	0.279	0.437	0.265	0.141	0.272	0.388	0.267	0.144
	Z	0.914	0.955	0.310	0.031	0.858	0.924	0.031	0.039	0.895	0.945	0.165	0.034	0.889	0.941	0.169	0.035
4.3	X	0.957	0.978	−0.651	0.020	0.572	0.728	−1.362	0.051	0.839	0.912	−0.937	0.025	0.789	0.873	−0.983	0.032
	Y	0.602	0.751	0.508	0.125	0.677	0.808	0.145	0.065	0.638	0.779	0.311	0.087	0.639	0.779	0.321	0.092
	Z	0.959	0.979	0.311	0.026	0.824	0.904	0.008	0.042	0.899	0.947	0.152	0.035	0.894	0.943	0.157	0.034
5	X	0.962	0.980	−0.599	0.017	0.867	0.929	−1.429	0.039	0.847	0.917	−0.949	0.024	0.892	0.942	−0.992	0.027
	Y	0.726	0.841	0.514	0.068	0.867	0.929	0.090	0.044	0.777	0.874	0.279	0.053	0.790	0.881	0.294	0.055
	Z	0.973	0.987	0.385	0.024	0.923	0.960	0.032	0.030	0.935	0.967	0.194	0.028	0.944	0.971	0.204	0.028
6	X	0.975	0.987	−0.507	0.024	0.909	0.952	−1.484	0.050	0.952	0.975	−0.979	0.018	0.945	0.972	−0.990	0.031
	Y	0.675	0.806	0.553	0.108	0.876	0.934	0.044	0.061	0.779	0.876	0.257	0.077	0.777	0.872	0.285	0.082
	Z	0.991	0.995	0.341	0.012	0.915	0.956	−0.022	0.050	0.949	0.974	0.156	0.032	0.951	0.975	0.158	0.031
6.5	X	0.959	0.979	−0.499	0.032	0.805	0.892	−1.522	0.067	0.886	0.940	−0.989	0.033	0.883	0.937	−1.003	0.044
	Y	0.728	0.843	0.683	0.118	0.695	0.820	0.043	0.061	0.572	0.728	0.314	0.080	0.665	0.797	0.347	0.086
	Z	0.995	0.998	0.527	0.010	0.946	0.972	0.025	0.033	0.942	0.970	0.243	0.028	0.961	0.980	0.265	0.024
7	X	0.604	0.753	0.831	0.086	0.910	0.953	−0.982	0.114	0.596	0.747	−0.079	0.100	0.703	0.818	−0.077	0.100
	Y	0.950	0.974	3.199	0.087	0.984	0.992	−0.343	0.024	0.933	0.965	1.060	0.032	0.956	0.977	1.305	0.048
	Z	0.985	0.992	0.966	0.032	0.777	0.875	−0.214	0.039	0.978	0.989	0.267	0.021	0.913	0.952	0.340	0.031
8	X	0.921	0.959	0.852	0.054	0.363	0.532	−1.056	0.192	0.322	0.487	−0.109	0.116	0.536	0.660	−0.104	0.121
	Y	0.988	0.994	3.453	0.037	0.963	0.981	−0.478	0.029	0.834	0.910	1.065	0.014	0.929	0.962	1.347	0.027
	Z	0.997	0.998	0.793	0.025	0.954	0.976	−0.339	0.054	0.989	0.995	0.162	0.024	0.980	0.990	0.205	0.035
9	X	0.906	0.951	0.981	0.071	0.848	0.918	−1.004	0.047	0.868	0.929	−0.049	0.043	0.874	0.933	−0.024	0.054
	Y	0.980	0.990	3.536	0.067	0.987	0.993	−0.498	0.013	0.780	0.877	1.088	0.028	0.916	0.953	1.375	0.036
	Z	0.999	1.000	1.097	0.017	0.812	0.897	−0.398	0.058	0.996	0.998	0.254	0.018	0.936	0.965	0.318	0.031
10	X	0.931	0.964	0.927	0.075	0.652	0.790	−1.176	0.208	0.104	0.188	−0.128	0.120	0.562	0.647	−0.126	0.134
	Y	0.987	0.994	3.452	0.087	0.957	0.978	−0.494	0.056	0.863	0.926	1.045	0.027	0.936	0.966	1.335	0.057
	Z	0.990	0.995	1.169	0.052	0.833	0.909	−0.391	0.074	0.992	0.996	0.280	0.022	0.938	0.967	0.352	0.049
	Average	0.845	0.896		0.055	0.776	0.857		0.062	0.757	0.836		0.047	0.793	0.863		0.055

ICC = Interclass correlation coefficient; g = acceleration in gravitational force equivalent; SD = standard deviation.

**Table 2:** ICC values and standard deviation grouped by speed (km/h) and axis, in thigh position.

ICC THIGH 95%		Mean Peaks Values				Mean Minimum Values				Mean Values				By Rows			
Km/h	Axis	Single ICC	Average ICC	Average Peaks (g)	SD (g)	Single ICC	Average ICC	Average Valleys (g)	SD (g)	Single ICC	Average ICC	Average (g)	SD (g)	Mean Single ICC	Mean Average ICC	Mean Average (g)	Mean SD (g)
1.4	X	0.585	0.738	0.166	0.074	0.308	0.471	−0.261	0.093	0.535	0.697	−0.047	0.077	0.476	0.636	−0.048	0.081
	Y	0.852	0.920	−0.839	0.006	0.144	0.251	−1.211	0.026	0.767	0.868	−0.988	0.006	0.588	0.680	−1.013	0.013
	Z	0.792	0.884	0.391	0.039	0.966	0.983	−0.277	0.019	0.950	0.975	−0.073	0.022	0.903	0.947	0.014	0.027
2.9	X	0.874	0.933	0.439	0.060	0.953	0.976	−0.542	0.050	0.837	0.911	−0.071	0.046	0.888	0.940	−0.058	0.052
	Y	0.284	0.442	−0.492	0.024	0.953	0.976	−1.589	0.046	0.453	0.624	−1.000	0.011	0.563	0.680	−1.027	0.027
	Z	0.915	0.956	0.782	0.080	0.945	0.972	−0.442	0.039	0.924	0.961	−0.051	0.023	0.928	0.963	0.096	0.048
4.3	X	0.904	0.950	0.613	0.080	0.940	0.969	−0.798	0.066	0.781	0.877	−0.077	0.046	0.875	0.932	−0.088	0.064
	Y	0.951	0.975	−0.274	0.029	0.838	0.912	−1.819	0.050	0.865	0.928	−1.030	0.006	0.885	0.938	−1.041	0.028
	Z	0.882	0.937	1.155	0.145	0.952	0.975	−0.593	0.045	0.951	0.975	−0.061	0.021	0.928	0.962	0.167	0.070
5	X	0.740	0.850	0.753	0.096	0.979	0.989	−0.722	0.034	0.907	0.951	−0.010	0.039	0.875	0.930	0.007	0.056
	Y	0.813	0.897	−0.128	0.045	0.882	0.937	−1.950	0.059	0.731	0.844	−1.048	0.009	0.808	0.893	−1.042	0.038
	Z	0.660	0.795	1.311	0.180	0.967	0.983	−0.707	0.051	0.885	0.939	−0.045	0.022	0.838	0.906	0.186	0.085
6	X	0.862	0.926	0.847	0.076	0.883	0.938	−0.919	0.072	0.946	0.972	−0.095	0.028	0.897	0.945	−0.056	0.058
	Y	0.958	0.979	0.035	0.033	0.950	0.974	−2.290	0.056	0.892	0.943	−1.070	0.006	0.933	0.965	−1.109	0.032
	Z	0.656	0.792	1.486	0.227	0.969	0.984	−0.891	0.051	0.878	0.935	−0.081	0.031	0.834	0.904	0.171	0.103
6.5	X	0.979	0.989	0.967	0.062	0.857	0.923	−0.853	0.061	0.936	0.967	−0.059	0.034	0.924	0.960	0.018	0.052
	Y	0.972	0.986	0.168	0.034	0.967	0.983	−2.501	0.055	0.946	0.973	−1.068	0.005	0.962	0.981	−1.134	0.031
	Z	0.624	0.768	1.281	0.220	0.938	0.968	−1.079	0.067	0.846	0.917	−0.179	0.038	0.802	0.884	0.008	0.109
7	X	0.965	0.982	0.865	0.060	0.882	0.937	−0.986	0.123	0.816	0.899	−0.091	0.054	0.887	0.939	−0.071	0.079
	Y	0.893	0.943	0.397	0.044	0.976	0.988	−2.788	0.030	0.615	0.762	−1.099	0.015	0.828	0.898	−1.163	0.030
	Z	0.807	0.893	1.098	0.164	0.911	0.953	−1.066	0.068	0.853	0.921	−0.064	0.036	0.857	0.922	−0.011	0.089
8	X	0.934	0.966	0.988	0.097	0.545	0.705	−1.010	0.180	0.472	0.641	−0.089	0.111	0.650	0.771	−0.037	0.129
	Y	0.950	0.974	0.646	0.062	0.986	0.993	−2.909	0.040	0.881	0.937	−1.113	0.017	0.939	0.968	−1.125	0.040
	Z	0.833	0.909	1.181	0.156	0.998	0.999	−1.231	0.013	0.824	0.904	−0.127	0.042	0.885	0.937	−0.059	0.070
9	X	0.970	0.985	1.135	0.059	0.889	0.941	−1.129	0.083	0.048	0.091	−0.085	0.039	0.636	0.673	−0.027	0.060
	Y	0.955	0.977	0.768	0.062	0.942	0.970	−3.122	0.087	0.805	0.892	−1.158	0.018	0.900	0.946	−1.171	0.056
	Z	0.832	0.908	1.311	0.180	0.967	0.983	−1.349	0.053	0.737	0.848	−0.108	0.051	0.845	0.913	−0.048	0.095
10	X	0.978	0.989	1.202	0.046	0.953	0.976	−1.246	0.070	0.883	0.938	−0.072	0.037	0.938	0.968	−0.039	0.051
	Y	0.941	0.970	0.885	0.063	0.973	0.986	−3.266	0.065	0.827	0.905	−1.185	0.022	0.914	0.954	−1.189	0.050
	Z	0.909	0.952	1.421	0.171	0.965	0.982	−1.529	0.054	0.663	0.798	−0.143	0.051	0.846	0.911	−0.084	0.092
	Average	0.842	0.905		0.089	0.879	0.919		0.060	0.782	0.860		0.032	0.834	0.895		0.060

ICC = Interclass correlation coefficient; g = acceleration in gravitational force equivalent; SD = standard deviation.

**Table 3:** Mean of ICC values for “single measurements” option, “average measurements” option, an average of the signal and standard deviation (SD), for each axis and velocity in each position, wrist and thigh.

	Mean of “Single” ICC		Mean of “Average” ICC		Average (g)		Mean of SD (g)	
	Wrist	Thigh	Wrist	Thigh	Wrist	Thigh	Wrist	Thigh
Axis X	0.721	0.805	0.812	0.869	−0.624	−0.040	0.061	0.068
Axis Y	0.727	0.832	0.813	0.890	0.714	−1.101	0.069	0.034
Axis Z	0.930	0.867	0.963	0.925	0.225	0.044	0.033	0.079
1.4 km/h	0.609	0.656	0.731	0.754	−0.203	−0.349	0.042	0.040
2.9 km/h	0.549	0.793	0.660	0.861	−0.181	−0.330	0.076	0.042
4.3 km/h	0.774	0.896	0.865	0.944	−0.168	−0.321	0.053	0.054
5 km/h	0.875	0.840	0.932	0.910	−0.165	−0.283	0.036	0.060
6 km/h	0.891	0.888	0.940	0.938	−0.182	−0.331	0.048	0.064
6.5 km/h	0.836	0.896	0.905	0.942	−0.131	−0.370	0.051	0.064
7 km/h	0.857	0.857	0.916	0.920	0.523	−0.415	0.060	0.066
8 km/h	0.815	0.825	0.870	0.892	0.488	−0.407	0.061	0.080
9 km/h	0.908	0.794	0.950	0.844	0.556	−0.415	0.040	0.070
10 km/h	0.812	0.899	0.860	0.944	0.520	−0.437	0.080	0.064

ICC = Interclass correlation coefficient; g = acceleration in gravitational force equivalent; SD = standard deviation.

## Data Availability

R-Code designed and data of the present study could be analyzed: https://doi.org/10.6084/m9.figshare.19518796.v1 (accessed on 5 April 2022).

## Appendix A

R-Code designed and data of the present study could be analyzed:

https://doi.org/10.6084/m9.figshare.19518796.v1 (accessed on 5 April 2022).
